# The Contribution of Upper Body Movements to Dynamic Balance Regulation during Challenged Locomotion

**DOI:** 10.3389/fnhum.2018.00008

**Published:** 2018-01-26

**Authors:** Kim J. Boström, Tim Dirksen, Karen Zentgraf, Heiko Wagner

**Affiliations:** ^1^Department of Movement Science, University of Münster, Münster, Germany; ^2^Department of Movement Science and Training in Sports, Goethe University Frankfurt, Frankfurt, Germany

**Keywords:** postural control, dynamic balance task, challenged locomotion, balance regulation, hip and ankle strategies

## Abstract

Recent studies suggest that in addition to movements between ankle and hip joints, movements of the upper body, in particular of the arms, also significantly contribute to postural control. In line with these suggestions, we analyzed regulatory movements of upper and lower body joints supporting dynamic balance regulation during challenged locomotion. The participants walked over three beams of varying width and under three different verbally conveyed restrictions of arm posture, to control the potential influence of arm movements on the performance: The participants walked (1) with their arms stretched out perpendicularly in the frontal plane, (2) spontaneously, i.e., without restrictions to the arm movements, and (3) with their hands on their thighs. After applying an inverse-dynamics analysis to the measured joint kinematics, we investigated the contribution of upper and lower body joints to balance regulation in terms of torque amplitude and variation. On the condition with the hands on the thighs, the contribution of the upper body remains significantly lower than the contribution of the lower body irrespective of beam widths. For spontaneous arm movements and for outstretched arms we find that the upper body (including the arms) contributes to the balancing to a similar extent as the lower body. Moreover, when the task becomes more difficult, i.e., for narrower beam widths, the contribution of the upper body increases, while the contribution of the lower body remains nearly constant. These findings lend further support to the hypothetical existence of an “upper body strategy” complementing the ankle and hip strategies especially during challenging dynamic balance tasks.

## 1. Introduction

When humans try to maintain their equilibrium in a challenging balancing task, they unwittingly engage upper body parts including their trunk, arms, and head (Otten, 1999; Milosevic et al., 2011; Schärli et al., 2013). Especially when balancing across a wire or a narrow beam, they intuitively stretch out and move their arms (Honegger et al., 2013; Patel et al., 2014). Such dynamic movements are possible because the many interconnected body parts are regulated by a powerful postural control system that allows for maintaining balance even when highly challenging external mechanical perturbations (Rietdyk et al., 1999; Cenciarini and Peterka, 2006; Vennila and Aruin, 2011) or relevant changes of sensory information occur (Horak, 2006; Horlings et al., 2009). How does the human postural control system regulate multiple joints and muscles along the kinematic chain to stabilize the body in challenging balance situations (Krishnamoorthy et al., 2005; Scholz et al., 2012)?

A widespread and frequently cited concept of postural control dates back to Nashner and McCollum (1985), who postulated the existence of two distinct postural control mechanisms termed the *ankle strategy* and the *hip strategy*, which are engaged by the nervous system either separately or in combination to reposition the body's center of mass over the base of support and thus maintain equilibrium. The ankle and hip strategies are usually quantified with joint kinematics and muscle activation (Federolf et al., 2013), which describe the human body as, respectively, an inverted single or double pendulum stabilized by movements about, respectively, the ankle and hip joints. Which one of these strategies is predominant depends on the task, in particular on its difficulty (Horak and Macpherson, 1996; Winter et al., 1997). For tasks with relatively low requirements on balance regulation, such as quiet standing, the ankle strategy with movements solely around the ankle is assumed to be most crucial for maintaining balance, while for more difficult tasks, e.g., one-leg stance with eyes closed, or backwards translation of the platform, the hip strategy with significant joint torques in the hip joint is regarded to be predominant (Horak and Nashner, 1986; Winter et al., 1996; Park et al., 2004). Notably, movements of the upper body, in particular of the arms, are not taken into account by these two postural control strategies. From a researcher's perspective, such conceptual restriction is advantageous in so far as (1) the underlying models are kept simple and their variables are easier to control, and (2) the models can more straightforwardly be validated on constrained experimental conditions, e.g., altered vision in a moving virtual environment (Scholz et al., 2012).

Recent studies, however, suggest to extend the concept of postural control strategies by taking into account also upper body movements, such as bending the trunk or raising and swaying the arms (Hsu et al., 2007; Pinter et al., 2008; Kilby et al., 2015). This extended concept is supported by empirical evidence showing that movements between pelvis and shoulder (Hsu et al., 2007; Horlings et al., 2009), as well as arm movements (Milosevic et al., 2011; Scholz et al., 2012; Patel et al., 2014), have a relevant contribution to maintaining balance. In particular for perturbed or challenged balance regulation, coordinated upper body activity seems to support ankle and hip movements in bringing the center of mass (CoM) back over the base of support (McIlroy and Maki, 1995; Marigold, 2002; Roos et al., 2008). Also, the role of arm movements in balance control during a complex dynamical task such as gait initiation has been investigated (Yiou and Do, 2011). Beside balance, arm movements may also serve to propel the body forward (Yiou, 2005; Yiou and Schneider, 2007).

In line with these findings, we seek to strengthen the hypothesis that in addition to movements about the ankle and hip joints, the upper body joints (especially the arms) make a substantial and functionally consistent contribution to balance regulation during challenging dynamic balance tasks. This contribution may be viewed as the result of a hypothesized *upper body strategy* complementing the hip and ankle strategies in supporting postural control during challenging dynamic balance tasks.

Much of the existing evidence is based on kinematic approaches intended to analyze the joint angle variability in terms of angular velocities (Horlings et al., 2009; Honegger et al., 2013) and rotations (Pinter et al., 2008; Roos et al., 2008). These approaches provide useful insights into different joint movement patterns and strategies when performing balance tasks. However, balance is a dynamic process that depends on forces and torques, so that joint movements of the upper body might be influenced by anti-gravity forces generated by movements of the lower body. To evaluate the role of torques and forces, it is necessary to apply an inverse-dynamics analysis.

Also, many studies examine whole-body balance movements only for static balance tasks [quiet or perturbed standing (Creath et al., 2005; Hsu et al., 2007; Pinter et al., 2008; Federolf et al., 2013; Honegger et al., 2013; Wang et al., 2014)], or for normal walking (Herr and Popovic, 2008; Silverman et al., 2012). The demands for the balance regulation system during challenging dynamic balance tasks such as walking across a wire or a narrow beam, are considerably higher than for normal standing or walking, and an adequate description would be of high practical relevance, e.g., for sports and outdoor activities, but also to investigate on the falling risk of elderly people. For dynamic balance tasks it appears reasonable to assume that there are several strategies simultaneously engaged for the same task, since individually different, multi-joint coordination patterns occur (Bernstein, 1967; Alexandrov et al., 2005; Wang et al., 2014).

To address these issues, we applied an inverse-dynamics analysis to kinematic and force plate data recorded during a dynamic balance task involving challenged locomotion, to gain information about the extent to which the multiple joints of the entire body, and in particular of the upper body as compared to the lower body, contribute to postural control under such circumstances. As an extension of Nashner and McCollum's concept of ankle and hip strategies, we hypothesize that in a dynamic balance task during challenged locomotion, (1) a multi-joint coordination pattern with a significant contribution of upper body movements shows up, and (2) the contribution of upper body movements to balance regulation increases with task difficulty.

## 2. Materials and methods

### 2.1. Participants

Recruited by announcements via e-mail and notice board, 22 healthy males aged 15–28 years (24.27 ± 3.01 years) volunteered in the study. In a preceding interview, the participants had been asked for limitations or deficits in their cognitive and motor abilities, and in particular in postural control. Positive reports would have been taken as exclusion criteria, but were not given. We restricted our study to male subjects to avoid gender-dependent differences in gait patterns (Chumanov et al., 2008). The average body height was 1.83 ± 0.065 m and the average body mass was 78.14 ± 7.18 kg. The participants received no payment, and they had been informed beforehand about the procedure of the study. All participants gave written informed consent in accordance with the Declaration of Helsinki. In addition, one underage participant had their parents sign the consent. The data gained from the underage participant was later discarded due to potential, uncontrollable side-effects caused by the participant's potentially immature postural control system. The study was approved by the local ethics committee of the University of Münster “Ethikkommission des FB 7: Psychologie und Sportwissenschaft,” approval number: 2014-03-TD.

### 2.2. Experimental procedure

**Figure 1:**
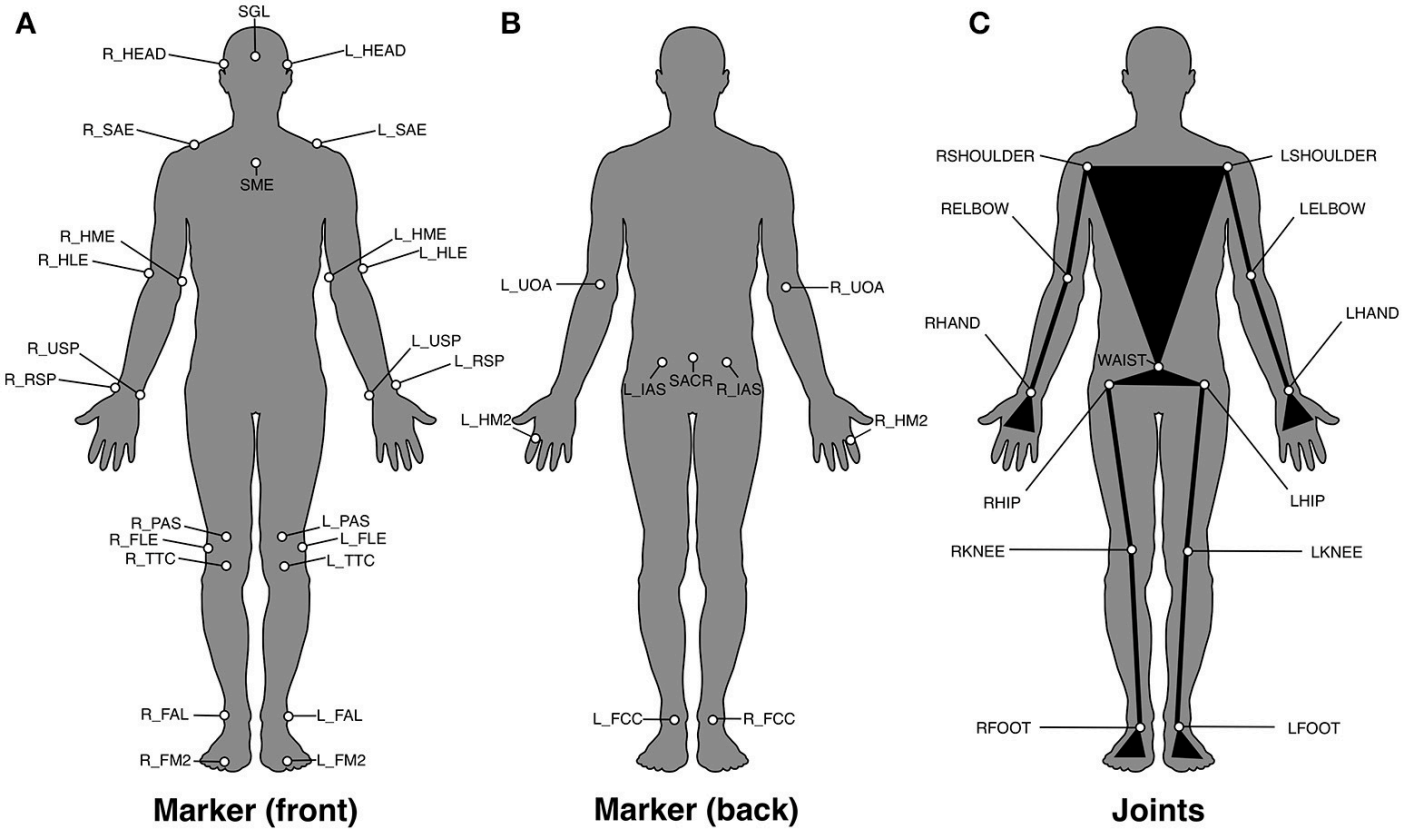
**(A,B)** Positioning and labeling of the markers attached to the participant's body according to Tranberg (2010). **(A)** Front view and **(B)** back view. **(C)** Positioning and labeling of the joints used for the inverse-dynamics analysis.

**Figure 2:**
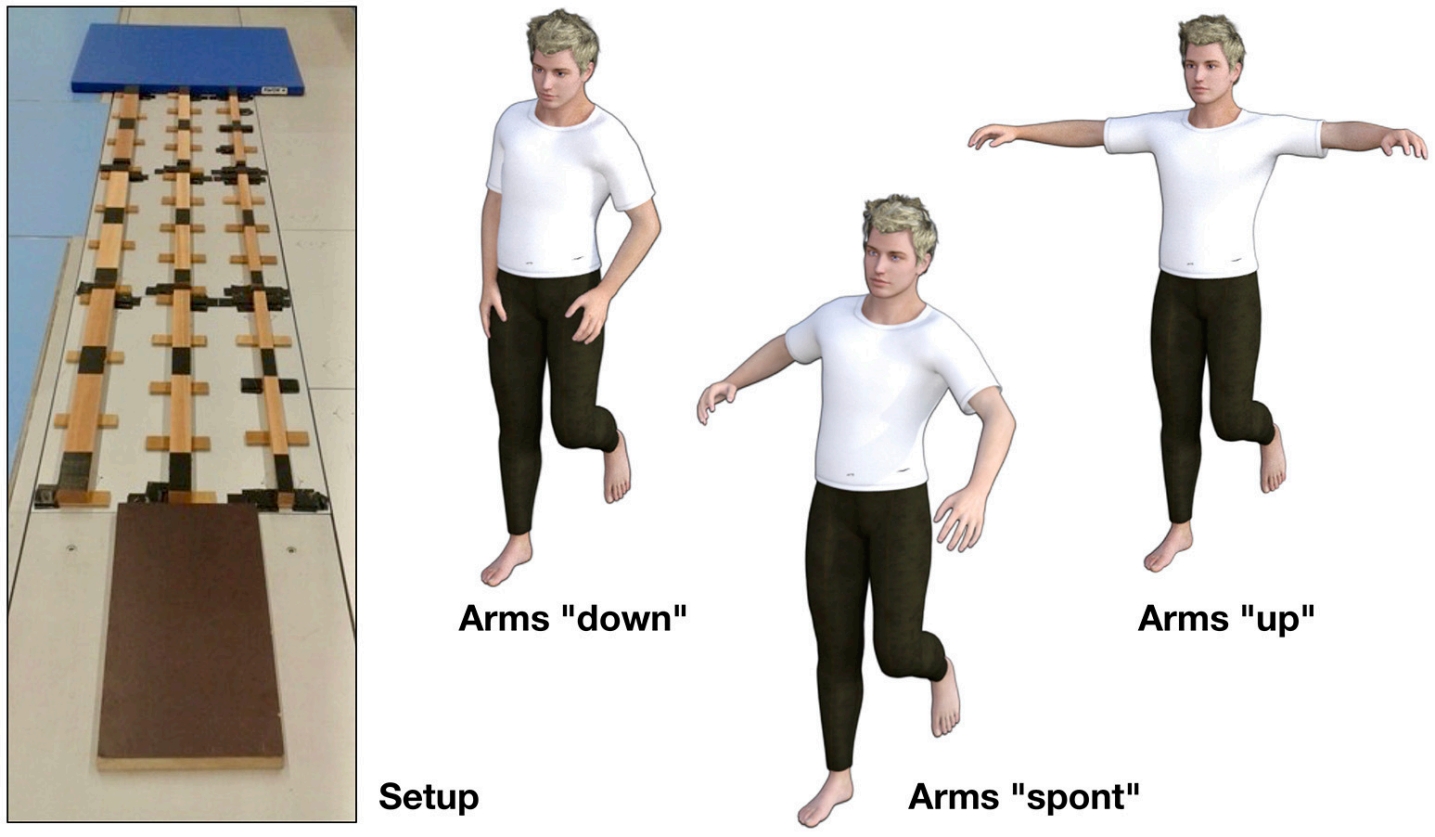
**Left:** Experimental setup. The balance beams were affixed to the force plates with double-sided tape. Additionally, black tape was used to visibly separate the regions assigned to the individual steps. Participants were asked to only step between the black tape marks. **Right:** Verbally instructed arm postures. On the “down” condition, participants had to keep their hands on their thighs, on the “up” condition they had to stretch out their arms perpendicularly in the frontal plane, and on the “spont” condition they could freely move their arms.

Participants stood barefoot on a panel 60 × 30 × 3 cm in size that was located directly in front of three balance beams (length 264 cm, height 3 cm) of different width (6, 4.5, and 3 cm). The three balance beams were affixed with tape in parallel across three stationary, successively arranged force plates (9287CA, 90 × 60 cm, Kistler Instrumente AG, Winterthur, Switzerland), to obtain ground reaction forces sampled at 800 Hz (**Figure 2** left). Right before each subject's experimental session, we checked the setup against a possible transfer of measured force across the force plates by controlling the position of the force vector when the subject stepped on the beam. The joint movements were captured by 33 reflective markers which were placed on specific skeletal landmarks of all major segments of the subject's body according to Tranberg (2010) (Figures 1A,B). Marker trajectories were recorded using a motion-capture system of 10 high-speed infrared cameras with a sampling rate of 200 Hz (Oqus 500, Qualisys AB, Goeteborg, Sweden). The force plates and the kinematic system were synchronized with an external analog trigger signal (Qualisys AB, Goeteborg, Sweden).

From a tandem stance position, participants were asked to perform six steps across each balance beam, starting with the right foot, and to complete three trials per measurement condition. Each measurement condition consisted of balancing over one out of three beams of varying width. Additionally, to control the potential influence of arm movements, we had the participants take three different arm postures during walking (Figure 2 right): (1) with their hands on their thighs, right below the hip (“down” condition), (2) without any restrictions to arm posture (“spont” condition), and (3) with their arms stretched out at an angle of about 90° shoulder abduction in the frontal plane (“up” condition). Subsequent to determining the sequence of beam widths in a random order, the arm posture instructions were randomized. Compliance to the instructions was visually monitored, minor arm movements on the “up” and “down” condition were admitted, else the trial was aborted and repeated.

Furthermore, to reduce uncontrolled variability, the step frequency was dictated by a metronome at a frequency of 0.5 Hz, which turned out to be a comfortable frequency for the given tasks. Also, to reduce uncontrolled variability, and due to limitations imposed by the length of the force plates and the fact that the inverse-dynamics algorithm requires the participant to not step between the force plates, the step length was restricted to about 13 cm, which had been estimated on basis of the force plate length and a generic foot length of about 30 cm. The regions corresponding to the individual steps were marked on the balance beams with black tape, and the participants were asked to only step between the black regions. Between trials, the participants were allowed to recover for one minute by stepping down from the beams.

### 2.3. Data analysis

#### 2.3.1. Data preparation

Only trials in which the subject did not leave the verbally instructed arm posture (in the case of the “up” and “down” conditions), and did not leave the balance beam, were considered successful and were analyzed. The duration between the first and the last step was determined from the temporal distance between the maximum values of the horizontal component of the ground reaction force at the first and last step on the balance beam. The 3D-positions of the reflective markers (Figures 1A,B) were manually processed using the motion tracking software (Qualisys, Sweden), closing gaps whose length did not exceed 10 samples corresponding to 50 ms. Only completely reconstructed marker trajectories have been considered as valid and have been further processed. Due to measurement-related problems, the data from 6 subjects contained irreparable gaps and were excluded from analysis. In total, with the data from the underage participant also being discarded (see above), there were 15 participants whose data entered the final analysis.

#### 2.3.2. Inverse-dynamics analysis

We used a kinematic model with 14 body segments (feet, lower legs, thighs, pelvis, thorax, upper arms, forearms and hands) in Visual3D™ (C-Motion, Rockville, USA) and calculated the joint kinematics from the marker trajectories according to the joint coordinate system approach by Grood and Suntay (1983). Since the head was not integrated in the kinematic model used by Visual3D, we could not include the head in our calculations for the upper body joint group. The obtained joint kinematics and the simultaneously measured 3D ground reaction forces were low-pass filtered with a 15 Hz fourth-order zero-phase Butterworth filter. The kinematic data and the force plate data were then processed in Visual3D by using a conventional inverse-dynamics approach to determine the torques acting on each joint. The resulting time series of the force torques had the same sample rate as that of the kinematic data (200 Hz), which corresponds to a time step of Δ*t* = 5 ms. Note that the algorithm used in the Visual3D software for calculating the joint torques makes use of the measured ground reaction forces for all joints, including the upper body joints, even though the latter are only indirectly connected to the ground.

To successfully perform the inverse-dynamics analysis on the joint kinematics and the data from the force plates, it is technically necessary that there is no more than one foot on a force plate at a time, otherwise the analysis would not yield reliable results. Therefore, by visual inspection of the kinematic and force plate data, we manually split each force plate recording into two parts, each one containing one and a half steps, so that only one foot is on a force plate. We removed the intermediate phase of each part where one foot is on two plates at a time, so the two parts were not contingent. Since this lack of contingency would generate discontinuities when the data were merged again afterwards, we analyzed each of the two parts separately as follows.

The torque trajectories resulting from the inverse dynamics analysis were filtered and interpolated using a Savitzky-Golay filter (Savitzky and Golay, 1964) with a polynomial degree of 4 and a window size of 25 samples corresponding to 125 ms (using the MatLab function mssgolay). From these torque trajectories, the torque amplitude and variation have been calculated. Each of the two parts stemming from the splitting of the data outlined above yielded two corresponding values for the torque amplitude and variation (one for each part), and these value pairs were averaged into single values. With these values we performed the final statistical analysis described below.

#### 2.3.3. Torque variation and torque amplitude

The torque variation, or rather its square, the torque variance, has already been used in the literature as a measure of postural control activity (Andersson et al., 2002; Fransson et al., 2007a,b). It corresponds to the energy transferred from the body to the supporting surface (Magnusson et al., 1990). In addition to the torque variation, we also considered the torque *amplitude*, because we believe it relates to a different aspect of postural control activity. While the average torque amplitude of a given joint (Equation 3 below) directly quantifies the *amount of torque* that is applied to the joint on average within the observed time interval, the average torque variation (Equation 4 below) quantifies the *amount of change of the torque*, on average within the observed time interval. The energy consumption of a muscle depends not only on the mechanical work, which is generated during concentric contraction, but also on the muscles' heat production, which also occurs during isometric and eccentric contraction (Hill, 1938). Therefore, the average torque amplitude of a given joint group (Equation 5 below) is rather related to the *metabolic effort* per joint, of operating the joints in the group, which includes not only phases of dynamic movement but also phases of constant torque, e.g., while keeping a static posture. On the other hand, the average torque variation of the joint group (Equation 6 below) depends on the *rate of change* of the joint torques, and so it captures the dynamic aspects of postural control, and, therefore, is interpreted here as a measure of the *dynamic control* of the joints in the group.

**Figure 3:**
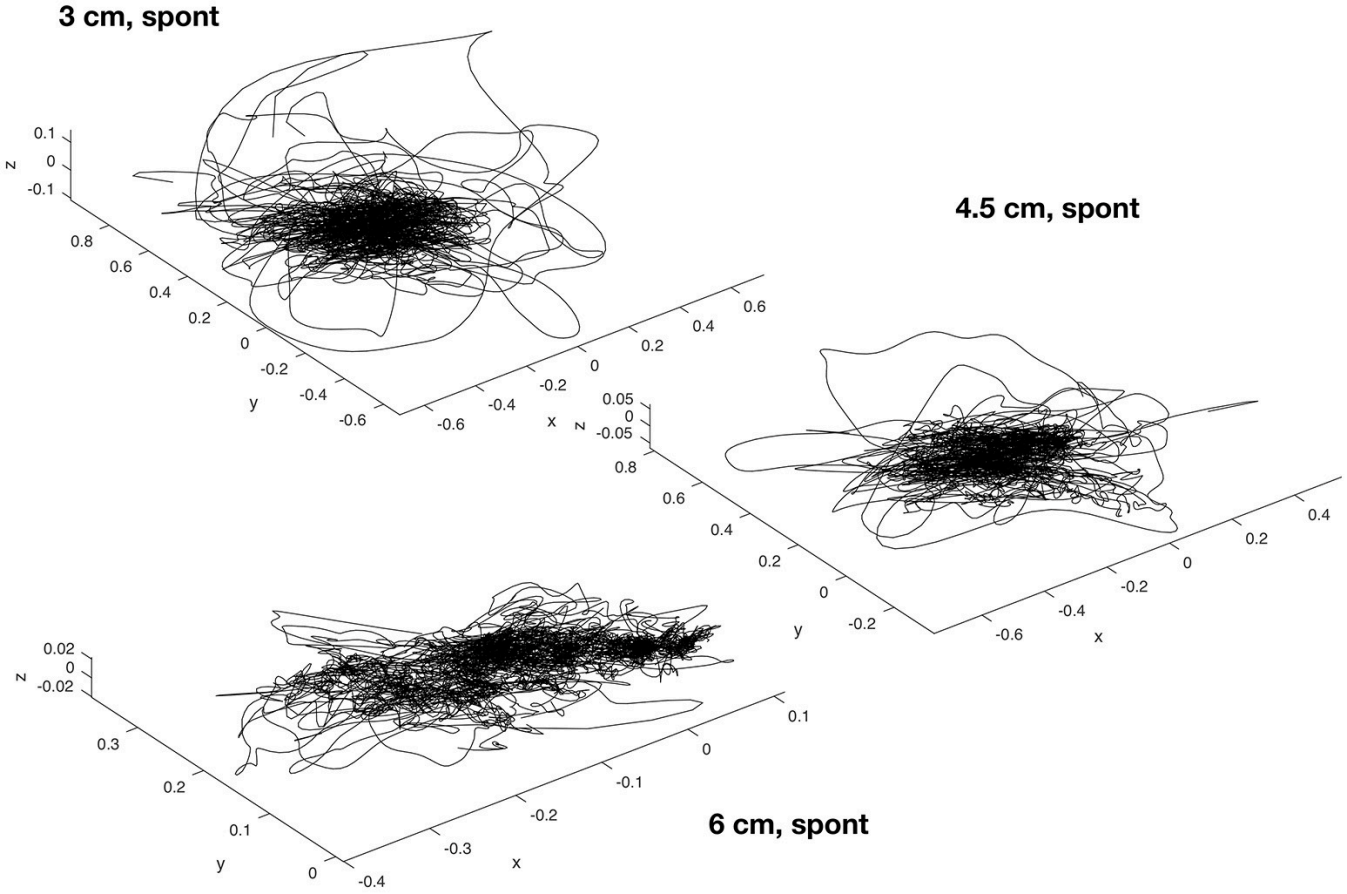
Sample plot of the torque trajectories of the right elbow joint of all 15 subjects, for varying beam width (3, 4.5, and 6 cm) and on the “spont” condition where the arms could freely be moved. Note how the torque space becomes increasingly exhausted with increasing task difficulty, that is, with decreasing beam width. The torque values are normalized to each subject's body weight and height, and are given in units of 1,000 Nm/(kg m). The entire dataset includes 13 joints on 9 conditions, which would yield 117 figures like those above.

The inverse-dynamics analysis yields a discrete time series of torques acting on the joints depicted in Figure 1C. (See Figure 3 for an illustration of the trajectory data). For each of these joints *j* there are three rotational degrees of freedom of its torque, forming a three-dimensional vector **τ**_*j*_(*t*), where *t* = 1, …, *T* is the discrete time index indexing *T* samples. In line with Fransson et al. (2007a,b), we reduced the inter-subject variability caused by the individual influence of body mass and body weight on the torque by normalizing it accordingly, obtaining the *specific* torque (torque per kilogram and meter),

(1) $$\begin{array}{r} {\tilde{\tau }}_{j}\left(t\right)=\frac{\tau_{j}\left(t\right)}{m_{s}\cdot l_{s}}, \end{array}$$

where *m*_*s*_ and *l*_*s*_ are the subject's body mass and height, respectively. For simplicity, we keep using the term “torque” in the following, although referring to the specific torque defined above. Based on the specific torque, we considered the *instantaneous torque amplitude* acting on each joint *j* at time *t*, defined by

(2) $$\begin{array}{r} a_{j}\left(t\right)=ǁ{\tilde{\tau }}_{j}\left(t\right)ǁ, \end{array}$$

where ǁ · ǁ is the usual Euclidean vector norm, defined for any given 3d-vector $v={\left(v_{1},v_{2},v_{3}\right)}^{T}$ as $ǁvǁ=\sqrt{v_{1}^{2}+v_{2}^{2}+v_{3}^{2}}$. Based on the instantaneous torque amplitude, we considered the *average torque amplitude* of a given joint *j* as the temporal mean

(3) $$\begin{array}{r} a_{j}=\frac{1}{T}\overset{T}{\underset{t=1}{\sum }}a_{j}\left(t\right), \end{array}$$

and the *average torque variation* of a given joint *j* as the temporal standard deviation

(4) $$\begin{array}{r} v_{j}=\sqrt{\frac{1}{T-1}\overset{T}{\underset{t=1}{\sum }}{\left(a_{j}\left(t\right)-a_{j}\right)}^{2}}. \end{array}$$

**Figure 4:**
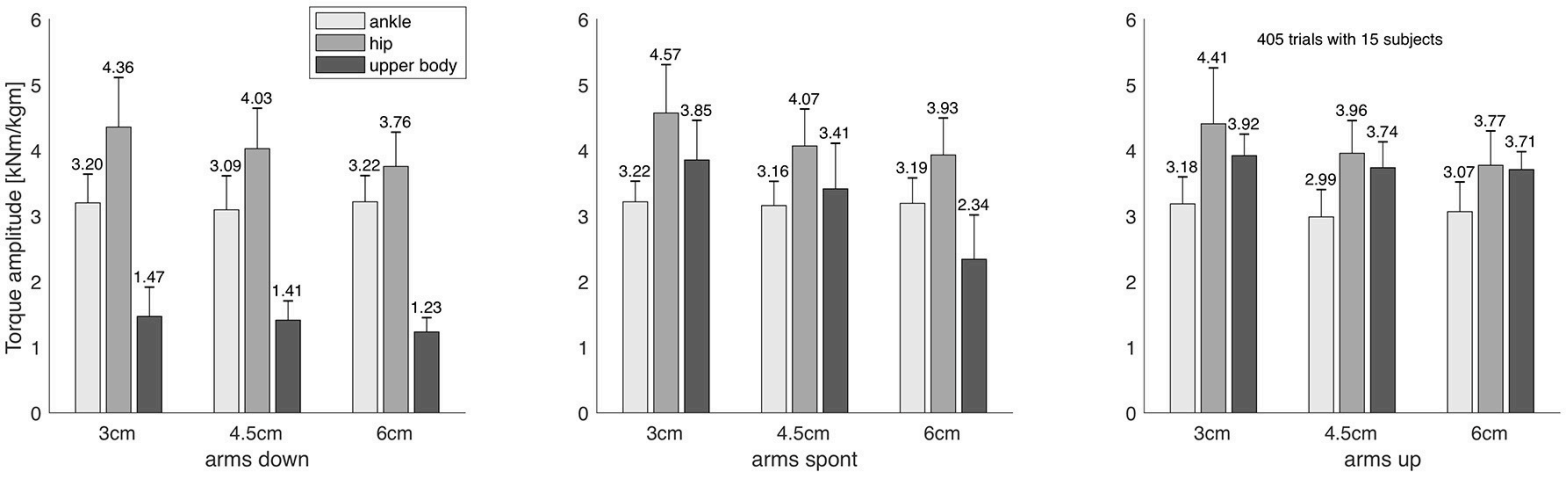
Average group torque amplitude of the three joint groups ‘ankle,’ ‘hip,’ and ‘upper body,’ as defined in Equation 5, displayed for all nine combinations of measurement conditions. Error bars indicate the standard deviation, values are normalized to each subject's body weight and height, and are given in units of 1,000 Nm/(kg m).

**Figure 5:**
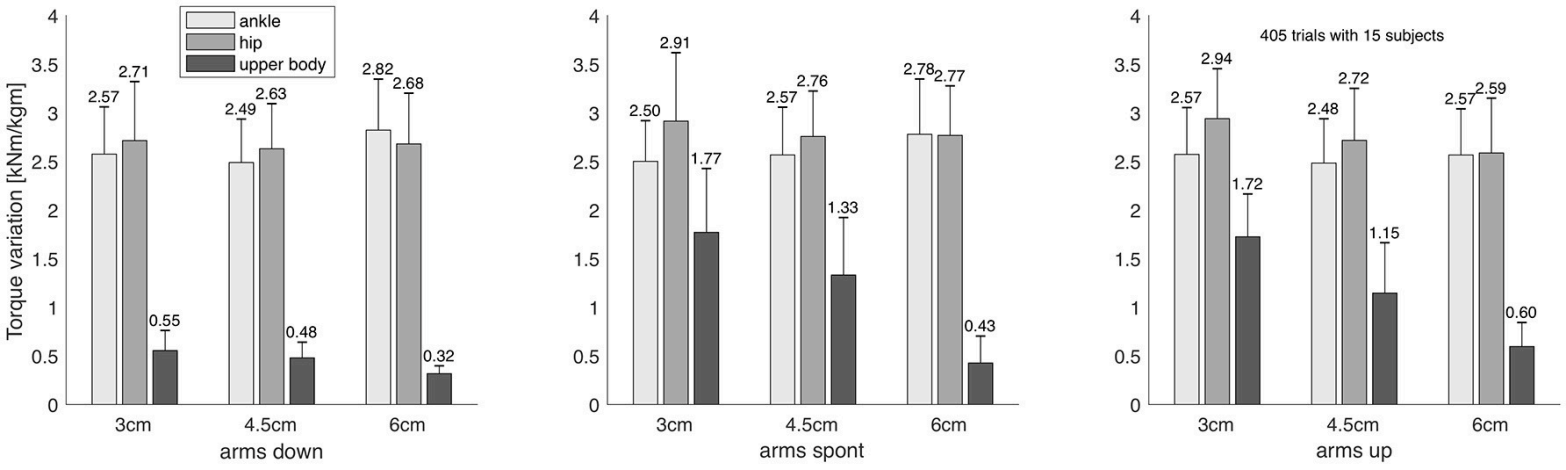
Average group torque variation of the three joint groups ‘ankle,’ ‘hip,’ and ‘upper body,’ as defined in Equation 6, displayed for all nine combinations of measurement conditions. Error bars indicate the standard deviation, values are normalized to each subject's body weight and height, and are given in units of 1,000 Nm/(kg m).

Finally, we considered the *average group torque amplitude* of a given joint group *J* = {*j*_1_, *j*_2_, …} as the group average

(5) $$\begin{array}{r} a\left(J\right)=\frac{1}{\left|J\right|}\underset{j\in J}{\sum }a_{j}, \end{array}$$

and the *average group torque variation* of joint group *J* as the group average

(6) $$\begin{array}{r} v\left(J\right)=\frac{1}{\left|J\right|}\underset{j\in J}{\sum }v_{j}, \end{array}$$

where |*J*| is the number of joints in *J*. Hence, *a*(*J*) and *v*(*J*) are the average torque amplitude and variation *per joint in the group*, respectively. As outlined above, these two measures are central to our analysis and are shown in Figures 4, 5, respectively. For simplicity, we will use the terms “torque amplitude” and “torque variation” to refer to the average group torque amplitude and variation, respectively.

#### 2.3.4. Statistics

Torque amplitude and variation have been calculated for 16 subjects, on nine experimental conditions, and for three joint groups “ankle,” “hip,” and “upper body,” defined in Table 1. Each of the nine experimental conditions results from a combination of one out of three beam widths (3 cm, 4.5 cm, and 6 cm) and one out of three specified arm postures (‘down,’ ‘spont,’ and ‘up,’ see Figure 2). For each of the two dependent variables “torque amplitude” and “torque variation” we performed a separate three-factor ANOVA with the factors “beam width,” “arm posture,” and “joint group,” yielding information about which of the factors have a significant influence on the respective dependent variable. Significance and effect size are reported as *p*-value and partial eta squared ($\eta_{p}^{2}$), respectively. The significance level has been set to α = 0.05, so that *p* < 0.05 indicates a significant effect. More precisely, the *p*-value equals the probability of observing an effect size as or more extreme assuming the null hypothesis was true. Of a given factor, the effect size partial eta squared equals the variance explained by that factor, divided by the variance remaining after excluding the variance explained by the other factors.

**Table 1:** Definition of the joint groups, with the group names and the associated joints, whose positions are defined in Figure 1C.

**Joint group**	**Joints**
Upper body	LSHOULDER, RSHOULDER, LELBOW, RELBOW, LHAND, RHAND, WAIST
Hip	LHIP, RHIP
Ankle	LFOOT, RFOOT

*The knees were included in the inverse-dynamics analysis, but where not part of any of the analyzed joint groups*.

It is often desirable to make a *post-hoc* pairwise multiple-comparison of individual results to test for significant differences. Quite generally, a “*post-hoc* test” is a test of a hypothesis that was not specified before seeing the data. In the ubiquitous case of multiple *post-hoc* tests, a statistical correction is necessary to reduce the risk of obtaining false positives. Typically, *post-hoc* tests involve the pairwise comparison of values after having performed an ANOVA on these values. In the present case, however, a pairwise comparison of the measured torque amplitudes and variations does not serve our scientific objective. Rather, in view of our main hypothesis 2, we are interested in the *correlation* between beam width on one side, and torque amplitude and variation on the other side: Does the contribution of upper body movements to balance regulation, measured in terms of torque amplitude and torque variation of upper body joints, in fact increase with task difficulty, measured in terms of beam width? Hence, it is straightforward to perform a *post-hoc correlation* test, instead of a *post-hoc* multiple-comparison test, to reveal significant correlations between the beam width as the independent variable, and torque amplitude and variation as the dependent variables, conditional on the factors “arm posture” and “joint group.” To this aim, we determined Pearson's correlation coefficient *r* indicating the strength and sign of the correlation, together with the corresponding *p*-values indicating the significance of the correlation. All *p*-values of the *post-hoc* analysis have been statistically corrected using the Bonferroni-Holm method.

The effect sizes corresponding to partial eta squared, $\eta_{p}^{2}$, and the correlation coefficient, *r*, have been rated as “small,” “medium,” and “large” using a widespread rating scheme proposed by Cohen (1988, 1992), also known as *Cohen's rule of thumb* (Table 2). Note that, unlike with significance levels, there is no strict, generally agreed convention for the rating of effect sizes, and also Cohen's rule of thumb must be used with caution. Since there are no sharp limits between the rating categories, we report in-between values as “small to medium” and so on, and we denote values substantially greater than the value rated by Cohen as “large,” as “very large.” Effects whose *p*-value is greater than *p* > 0.05 are considered as non-evidenced and are not reported.

**Table 2:** Effect size rating according to Cohen's rule of thumb (Cohen, 1988, 1992), for the absolute value of the correlation coefficient *r* and partial eta squared $\eta_{p}^{2}$.

	**Small**	**Medium**	**Large**
|**r**|	0.10	0.30	0.50
$\eta_{p}^{2}$	0.01	0.06	0.14

## 3. Results

We found that when the arms were not restricted to be held down, the upper body joints contribute substantially to balance regulation in the studied dynamic balance tasks, both with regard to torque amplitude (Figure 4) and variation (Figure 5). For illustration, Figure 3 exemplarily shows the torque trajectories of the right elbow joint of all 15 subjects for varying beam width and on the “spont” condition where the arms could freely be moved.

The ANOVA of the average torque amplitudes of the “ankle,” “hip,” and “upper body” joint groups, revealed highly significant, medium to large main effects (*p* < 0.0001) for all three factors ‘beam width’, ‘arm posture’, and ‘joint group’, as well as highly significant (*p* < 0.01), small to large interaction effects between these factors (Table 3). A similar picture emerges for the average torque variation, where the ANOVA revealed highly significant, medium to large main effects (*p* < 0.0001) for all three factors, as well as highly significant, small to large interaction effects (*p* < 0.01) between these factors (Table 4).

**Table 3:** ANOVA of the average group torque amplitudes displayed in Figure 4.

**Factor(s)**	***F***	***p***	**$\eta_{p}^{2}$**	**Effect size rating**
Beam width	*F*_(2, 386)_ = 23.88	<0.0001	0.11	Medium to large
Arm posture	*F*_(2, 386)_ = 85.77	<0.0001	0.31	Large
Joint group	*F*_(2, 386)_ = 222.33	<0.0001	0.54	Very large
Beam width ^*^ arm posture	*F*_(4, 386)_ = 2.81	<0.03	0.03	Small to medium
Beam width ^*^ joint group	*F*_(4, 386)_ = 5.62	<0.0003	0.06	Medium
Arm posture ^*^ joint group	*F*_(4, 386)_ = 87.36	<0.0001	0.48	Very large

*Interaction effects are denoted by an asterisk between the interacting factors, effect size is given by partial eta-squared, $\eta_{p}^{2}$. F-values and effect sizes have been rounded to two decimal places. Effect size was rated according to Cohen's rule of thumb (Table 2)*.

**Table 4:** ANOVA of the average group torque variations displayed in Figure 5.

**Factor(s)**	***F***	***p***	**$\eta_{p}^{2}$**	**Effect size rating**
Beam width	*F*_(2, 386)_ = 13.34	<0.0001	0.06	Medium
Arm posture	*F*_(2, 386)_ = 13.02	<0.0001	0.06	Medium
Joint group	*F*_(2, 386)_ = 583.14	<0.0001	0.75	Very large
Beam width ^*^ arm posture	*F*_(4, 386)_ = 3.39	<0.01	0.03	Small to medium
Beam width ^*^ joint group	*F*_(4, 386)_ = 15.14	<0.0001	0.14	Large
Arm posture ^*^ joint group	*F*_(4, 386)_ = 10.47	<0.0001	0.10	Medium to large

*Interaction effects are denoted by an asterisk between the interacting factors, effect size is given by partial eta-squared, $\eta_{p}^{2}$. F-values and effect sizes have been rounded to two decimal places. Effect size was rated according to Cohen's rule of thumb (Table 2)*.

The results of the *post-hoc* correlation analysis with the beam width as the controlled variable, and torque amplitude and variation as the observed variables, conditional on the factors “arm position” and “joint group” are given in Table 5.

**Table 5:** Analysis of the correlation between beam width as the controlled variable, and torque amplitude and variation as the observed variables, conditional on the factors “joint group” and “arm position”.

**Joint group**	**Arm posture**	***p***	**r**	**Effect size rating**
⋄ **TORQUE AMPLITUDE**				
Upper body	Spont	<0.0001	−0.69	Very large
⋄ **TORQUE VARIATION**				
Upper body	Spont	<0.0001	−0.72	Very large
Upper body	Down	<0.002	−0.53	Large
Upper body	Up	<0.0001	−0.75	Very large

*Negative values of r indicate a negative correlation of the observed variable with beam width, which amounts to a positive correlation with task difficulty. All 2 × 3 × 3 = 18 p-values resulting from this analysis have been statistically corrected using the Bonferroni-Holm method, only significant correlations (p < 0.05) are shown. Values for r have been rounded to two decimal places, |r| is interpreted as effect size and rated according to Cohen's rule of thumb (Cohen, 1988, 1992) (Table 2)*.

## 4. Discussion

The results confirm our main hypotheses that (1) there is a multi-joint coordination pattern with a significant contribution of upper body movements, and that (2) the contribution of upper body movements to balance regulation increases with task difficulty. These findings lend support to the hypothesized existence of an upper body strategy complementing the hip and ankle strategies in supporting balance regulation on challenging conditions (cf. Hsu et al., 2007; Pinter et al., 2008; Kilby et al., 2015).

In the following, we shall refer to the joints in the “upper body” group defined in Table 1 also as the “upper body joints,” and to the joints in the “hip” and “ankle” groups also as the “lower body joints.” Also, note that the beam width determines the difficulty of the balance task in a reciprocal manner, and note that verbally enforced arm posture restricts the range of possible movements and therefore the extent to which individual postural control strategies can be employed by the central nervous system. After these preliminaries, the results of our measurements are interpreted as follows:

When the participants were verbally enforced to keep their hands on the thighs, the torque amplitudes of the upper body joints were considerably smaller than those of the lower body joints (Figure 4). On the other hand, when the arms were not forced to be held down, the torque amplitudes of the upper and lower body were in the same ballpark. Under the latter circumstances, hence, the contribution of upper body joints to postural control during challenging dynamic balance tasks cannot be neglected. Moreover, the upper body torque amplitudes significantly increased with the difficulty of the task only when the arms could freely be moved, which can bee seen by looking at Figure 4, and by considering that the *post-hoc* correlation analysis revealed a strong anti-correlation between torque amplitude and beam width for the upper body joints only on the “spont” condition, while not revealing significant correlations on the “up” and “down” conditions (Table 3, upper section). The torque variation, on the other hand, showed a significant correlation with beam width across all arm postures. Taken together, these findings indicate that the upper body joints are increasingly engaged with increasing task difficulty, with the arms playing a crucial role.

In this context it seems logical that the torque amplitude and variation of the upper body joints were considerably smaller than the torque amplitudes and variations of the lower body joints when the participants had to keep their hands on their thighs (Figures 4, 5, first panel), indicating that the verbally enforced restriction of the arm posture leads to a reduced movement capacity of the arms, and consequently to smaller torque amplitudes and variations of the upper body joints. This shows in turn that arm movements contribute significantly to the generation of torques in the upper body, e.g., to brake an impending fall (Pijnappels et al., 2010). The results are in line with empirical evidence showing that coordinated upper body activity, particularly in challenged balance situations, is able to substantially support the lower body joints in keeping the center of mass over the base of support (McIlroy and Maki, 1995; Marigold, 2002; Roos et al., 2008).

As all experimental conditions had a highly significant, medium to large main effect on both the torque amplitude and variation (Tables 3, 4, respectively), it can be assumed that the joint groups contribute to both the metabolic effort and the dynamic control effort to varying degrees depending on the task difficulty and on the movement restrictions of the upper body. It is therefore interesting to look for interaction effects, and indeed we found highly significant interaction effects between all factors for both the torque amplitude and variation (Tables 3, 4). This means that the total effect depends on the specific combination of factors, and not only on the individual factors in isolation. In particular, the interaction of the factor “joint group” with the other two factors means that the three joint groups “ankle,” “hip,” and “upper body” behaved in a significantly different manner, which underpins the distinctness of the corresponding postural control strategies.

The torque amplitude of the upper body joints significantly and strongly decreased with beam width on the “spont” condition, and the torque variation showed the same behavior even across arm posture conditions (Figure 5, Table 5). There were no significant correlations for the lower body joint groups. Hence, with increasing task difficulty the central nervous system substantially increases the metabolic effort and the dynamic control effort for the upper body joints. This finding directly supports our main hypothesis 2 and indicates that the upper body strategy acts like a back-up strategy that is increasingly employed when balancing becomes more and more challenging. The lack of significant correlations for the lower body joint groups “hip” and “ankle” does not imply that there is no effect, but only that, if it exists, it is considerably smaller than for the upper body joints. This means that the metabolic effort and the dynamic control effort for the lower body joints is much less adapted to task difficulty than for the upper body joints.

As mentioned, the software used for the inverse-dynamics analysis had no kinematic model for the head, and therefore the latter could not be accounted for in our calculations. However, adding the neck joint to the upper body joint group should not qualitatively alter our main results, since doing so would potentially increase, rather than decrease, the contribution of the upper body, thereby strengthening the support for our hypothesis rather than weakening it.

Our findings are in line with the presumption of Hsu et al. (2007) that the coordinated activity of all body joints supports postural control to keep the center of mass (CoM) over the base of support and thus maintain equilibrium. Given the fact that the human body possesses more mechanical degrees of freedom than necessary to successfully perform a movement task (Latash, 2000), there is an infinite number of multi-joint movement solutions to achieve the task goal, forming an “uncontrolled manifold” which is exploited by the motor control system to successfully perform the given task in a stable, but at the same time flexible, way (Scholz and Schöner, 1999; Marigold, 2002; Pinter et al., 2008). The abundance of degrees of freedom allows for a certain amount of variability along the task-specific solution space that could be actively used to obtain higher balance accuracy by reducing the variability of crucial goal parameters, such as in the given balancing situation, the CoM position. In this context, Hsu et al. (2007) confirmed that movements in all major joints of the body are engaged in balance regulation. Similarly, Hof (2007) found that, especially when the base of support is getting smaller, a postural control strategy with the use of upper body movements is needed to generate sufficient anti-gravity forces to maintain postural stability. However, the mentioned studies examined segment movements during quiet and perturbed standing (static balance tasks), whereas we investigated multi-joint coordination patterns during challenged locomotion (dynamic balance task). Future studies may investigate the contribution of upper body movements to balance regulation in even more challenging dynamic balance situations, e.g., when adding external perturbations or changes of the sensory information. Based on the considerations above, we would predict that the contribution of upper body movements becomes even more pronounced.

## Author contributions

KZ, HW, and TD conceived the project and designed the experiments. TD was responsible for the data acquisition and conduction of the experiments. KB wrote the scripts, made the statistics, and prepared the figures. All authors interpreted the data and discussed the results. KB and TD wrote the manuscript. All authors approved the final version of the manuscript.

### Conflict of interest statement

The authors declare that the research was conducted in the absence of any commercial or financial relationships that could be construed as a potential conflict of interest. The reviewer ZP and handling Editor declared their shared affiliation.
